# Sedimentary signals of recent faulting along an old strand of the San Andreas Fault, USA

**DOI:** 10.1038/s41598-018-30622-3

**Published:** 2018-08-14

**Authors:** Julie C. Fosdick, Kimberly Blisniuk

**Affiliations:** 1 Department of Geography, University of Connecticut, Storrs, CT 06269 USA; 2 Center for Integrative Geosciences, University of Connecticut, Storrs, CT 06269 USA; 3 Department of Geology, San Jose State University, San Jose, CA 95192 USA

## Abstract

Continental transform fault systems are fundamental features in plate tectonics. These complex systems often constitute multiple fault strands with variable spatio-temporal histories. Here, we re-evaluate the complex history of the San Andreas Fault along a restraining bend in southern California (USA). The Mission Creek strand of the San Andreas Fault is a major geologic structure with ~90 km of strike-slip displacement but is currently mapped as inactive. Quaternary deposits record sediment dispersal across the fault from upland catchments and yield key markers of the fault’s displacement history. Our sediment provenance analysis from the Deformed Gravels of Whitewater and the Cabezon Fanglomerate provide detrital geochronologic and lithologic signatures of potential sources within the San Bernardino Mountains and Little San Bernardino Mountains. Statistical analysis shows that the Cabezon Fanglomerate is most compatible with the Mission Creek and Morongo Valley Canyon sources, rather than the Whitewater Canyon as previously suggested. We propose that displacement since deposition ~500–100 ka across the Mission Creek strand has separated these deposits from their original sources. These findings challenge the current paradigm that the Mission Creek strand is inactive and suggest that the fault continues to be a primary structure in accommodating deformation along the Pacific-North American plate boundary.

## Introduction

**Figure 1:**
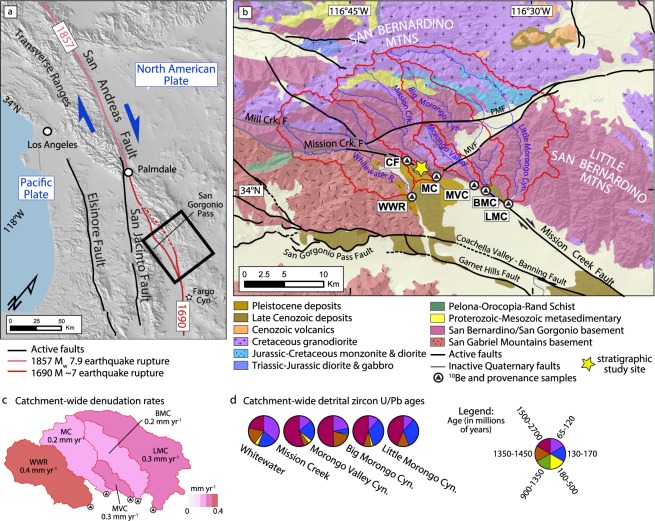
(**a**) Location map showing the Pacific-North American Plate boundary in southern California defined by the San Andreas Fault System and historic earthquake ruptures on the San Andreas Fault. The fault system comprises of the Elsinore, San Jacinto, and San Andreas faults. (**b**) Geologic map of the study area (area denoted by black box in part a) showing study site, modern drainage basins, faults, and representative bedrock geology source areas^6,18,28,29^. PMF = Pinto Mountain Fault; MVF = Morongo Valley Fault. Modern detrital sample locations: CF = Catclaw Flat; BMC = Big Morongo Canyon; LMC = Little Morongo Canyon; MC = Mission Creek; MVC = Morongo Valley Canyon; WWR = Whitewater River. (**c**) Catchment-wide denudation rates from modern catchments calculated from ^10^Be cosmogenic data. (**d**) Detrital zircon U/Pb ages from modern catchments. Base hillshade was generated with ESRI ArcMap v.10.4.1 software (under fair terms of use, https://www.esri.com/en-us/legal/copyright-trademarks) using a digital elevation model downloaded from the U.S. Geological Survey National Map database (https://viewer.nationalmap.gov)^53^.

Active tectonic plate boundaries exert first-order controls on topography, regional climate patterns, the routing of water and sediments from mountains to valleys, and the distribution of earthquakes^1–4^. Relative motion of the Pacific and North American plates is accommodated along multiple faults that make up the San Andreas Fault System^5,6^ (Fig. 1). Numerous geographic features that define the unique landscapes in the western U.S. can be attributed to this evolving plate boundary and its complex development since its inception ca. 27 Ma^7^. In southern California, some of the major tectonic events resulting from this plate boundary evolution include uplift and rotation of the Transverse Ranges, opening of the Gulf of California, and drainage reorganization of the Colorado River^3,6–10^. In such tectonically active settings, modification of topography requires study of long-term faulting and landform development to better understand modern plate motion. Today, natural hazards such as large-magnitude earthquakes and seismically triggered landslides pose extreme risk to > 18 million people in the densely populated region of southern California^11^. In this light, the southern San Andreas Fault is perhaps one of the most well-studied and monitored seismically active faults in the world^4,11,12^, and thereby offers an invaluable setting for studying continental transform fault systems through time.

Critical to understanding fault processes within strike-slip fault systems (i.e., strand switching, strain partitioning, temporal variability in deformation etc.)^13,14^ is recognizing both the timing and duration of fault activity along individual fault strands, and how strands accommodate total slip across the plate boundary^13–15^. Here, plate boundary deformation across the San Andreas Fault is partitioned into the Mill Creek, Mission Creek, Banning, Garnet Hill and the San Gorgonio Pass fault strands (Fig. 1b)^16–19^. Geologic and geodetic studies of the San Andreas Fault along its restraining bend near the San Gorgonio Pass (Fig. 1a) have long suggested a complex ca. 30 Ma history of fault reorganization and abandonment of its primary plate boundary faults, such as the Mill Creek and Mission Creek fault strands (Fig. 1b,c)^5,18,20,21^. This continuous fault evolution model suggests the Mill Creek and Mission Creek strands became inactive ~500 to 100 ka as plate boundary deformation migrated westward to other fault strands. Present kinematic models suggest that the majority of plate boundary deformation is localized onto the Banning, Garnet Hills and San Gorgonio Pass fault strands (Fig. 1c). These strands are therefore predicted by this model to take up most of the lateral displacement and therefore seismic hazard potential at this latitude^5,21–23^. In particular, the Banning strand through Coachella Valley is considered to be the locally active structure of the San Andreas Fault plate boundary.

In contrast, the Mission Creek fault strand (MCF) in the San Bernardino Mountains has accommodated at least ~90 km of dextral-oblique displacement of crystalline basement terranes since late Cenozoic time^20,21^, but is considered inactive at this location since Pleistocene time (~500–100 ka). This conclusion resulted from qualitative observations of buried Quaternary alluvium, the Cabazon fanglomerate, and from the apparent absence of displacement of these fanglomerates across the MCF^6,18,20,24^. These previous studies based on field observations of clast lithologies assumed fanglomerates along the foothills of the San Bernardino Mountains near San Gorgonio Pass were shed southward from the San Bernardino Mountains, or northward from the San Jacinto Mountains. The sedimentary provenance of these deposits, however, was uncertain because quantitative constraints using detrital zircon analysis to identify the sediment had not been done^6,18,20,24^.

Here, we propose a revision to the generally accepted fault paradigm that considers the MCF to be inactive since ~500–100 ka. We analyzed the sedimentary provenance of modern drainages within the San Bernardino Mountains and Little San Bernardino Mountains, along with Quaternary deposits found across the MCF to test this fault model. Our statistical analysis of detrital zircon U/Pb geochronology and conglomerate clast compositions suggest that the Cabazon Fanglomerate and younger Qt2 deposits correlate best to source areas across the MCF in the Mission Creek and Morongo Valley drainage basins (Fig. 1). This sediment ‘sink-to-source’ correlation thereby yields markers that define the long-term faulting history on the MCF and suggest more recent displacement since ~500–100 ka.

## Results

### Bedrock variability among modern drainage basins

The bedrock geology within the catchments of the San Bernardino and Little San Bernardino Mountains is sufficiently heterogeneous, with unique characteristic lithology and age groups to allow for a piercing point correlation along the MCF. The bedrock here consists predominantly of Mojave-type Precambrian gneiss intruded by Mesozoic plutons of the Sierra Nevada batholith^25,26^, with dominant lithologies consisting of biotite-gneiss, foliated granitoid, biotite quartz-monzonite, muscovite-garnet-granite, diorite, granodiorite, and amphibolite (Fig. 1c). The San Bernardino Mountains also preserve metasedimentary rocks of the Neoproterozoic Big Bear Group (Fig. 1d). In contrast, the Little San Bernardino Mountains consist of mostly Proterozoic gneiss (1.78 Ga Augen gneiss) and amphibolite with localized batholithic rocks, including a distinctive Jurassic diorite porphyry^25,27^. To the south across the MCF, the bedrock lithology consists of Cretaceous and older granitoid and gneissic rocks of San Gabriel Mountains-type^18,20,28,29^.

**Figure 2:**
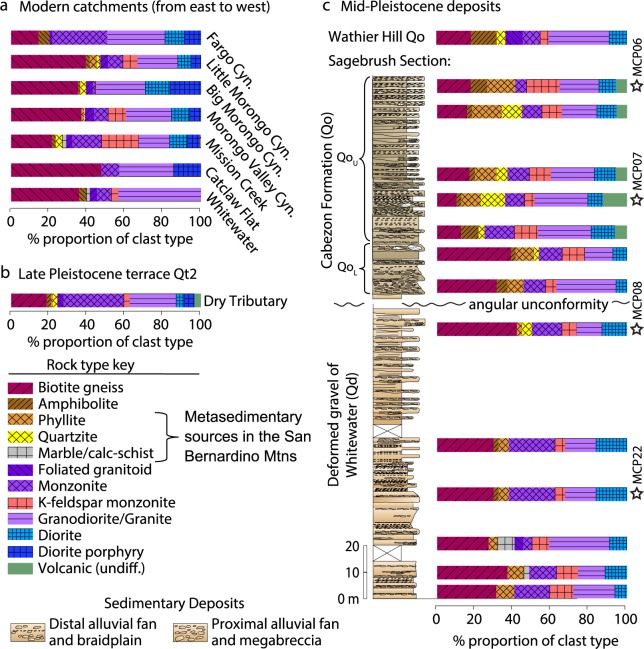
Sediment provenance and clast compositions from (**a**) modern catchments draining the San Bernardino and Little San Bernardino Mountains, (**b**) Late Pleistocene terrace fill, and (**c**) Mid-Pleistocene deposits measured in the Sagebrush Section and Wathier Hill. See Figs 1 and 4 for locations. Stars denote locations of detrital zircon U/Pb samples. The Cabezon Formation (Qo) and Qt2 terrace fill from Dry Tributary are compositionally most similar to Mission Creek, Morongo Valley Cyn., and Little Morongo Cyn., with diagnostic metasedimentary and diorite porphyry clast types.

Detrital samples from the modern catchments (Table S1), including Whitewater, Mission Creek, Morongo Valley, Big Morongo Valley, and Little Morongo Valley, that drain the San Bernardino Mountains and western Little San Bernardino Mountains show mostly igneous and high-grade metamorphic clasts with substantial variations in types and relative abundances of metasedimentary rock types^30^ (Fig. 2a, Table S2). Notably, Mission Creek, Big Morongo Valley, and Little Morongo Valley contain metasedimentary (quartzite, phyllite, and marble) clasts that are conspicuously lacking in Whitewater Canyon and Catclaw Flat (Fig. 2a). Diorite and diorite-porphyry are also more abundant in the western catchments. In comparison to these cobbles, the sand grain-size fractions (250–500 µm) from these catchments yield dominantly plutonic lithic grains, lesser components of metamorphic lithics, and abundant heavy minerals of amphibole, biotite, and pyroxene (Fig. S2). These findings are consistent with the modern distribution of bedrock lithology exposed in the modern catchments (Fig. 1b), although we note that the clast compositions reflect enrichment in more durable clast types (i.e., intrusives). Nonetheless, the close representation of diagnostic rock types allows us to distinguish between source areas and tack changing sediment compositions over time.

To evaluate the long-term catchment stability of the drainage divides and the potential for variability in erodibility among the Whitewater Canyon, Mission Creek, Morongo Valley, Big Morongo Valley, and Little Morongo Valley catchments over mid-Quaternary timescales, we determined catchment-wide denudation rates from modern stream deposits via cosmogenic ^10^Be concentrations. Denudation rates from these catchments range from 0.4 to 0.2 mm yr^−1^ (Fig. 1c), indicating relatively uniform erosion among the catchments along the MCF. These denudation rates are comparable within uncertainties (Table S6), though slightly lower, than exhumation rates derived from bedrock low-temperature apatite (U-Th)/He thermochronology data, which suggest long-term erosion rates of 0.4 to 0.6 mm yr^−1^ since 5–7 Ma in the San Bernardino Mountains and Little San Bernardino Mountains^31–33^. Taken together, these data support a relatively uniform erosional history across the catchments for tectonically active, arid settings, and validate the use of detrital mineral and sediment tracers for provenance comparison between the modern catchments and mid-Quaternary deposits^34,35^.

### Detrital geochronologic signatures of potential sources areas

**Figure 3:**
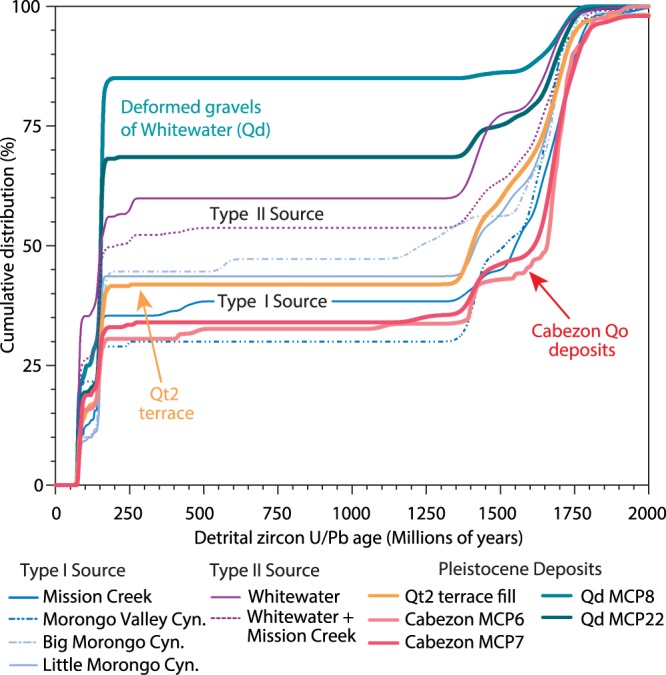
Detrital zircon U/Pb geochronology results showing cumulative age distributions for active catchments (Type I and Type II sources) and Pleistocene deposits. Both the Qt2 terrace fill and Cabezon fanglomerate are statistically equivalent to Type I sediment sources with a strong match to Mission Creek and Morongo Valley Cyn.

Uranium-Lead geochronology by laser ablation inductively coupled plasma mass spectrometry (U-Pb LA-ICP-MS) on detrital zircon crystals collected from the modern catchments and Pleistocene sedimentary deposits reveal distinct crystallization age components that we use to test provenance relationships (Fig. 3). Modern stream deposits that represent an integrated catchment-wide source yield five primary zircon U/Pb age groups in varying abundances: Mesozoic ages: 65–120 Ma, 130–170 Ma, Paleozoic Ages: 180–500 Ma, and Proterozoic ages: 950–1350 Ma, 1350–1450 Ma, and 1500–2700 Ma (Fig. 1d), with minor age components of 900–1350 Ma and 2.0–2.7 Ga. These primary age groups correspond to the Mesozoic plutons of the Sierra Nevada batholith, and to the Paleozoic and Proterozoic Mojave-type basement^27,36^. We performed a Pearson Chi-squared statistical analysis of detrital zircon age categories to evaluate if zircon ages analyzed from modern catchments adequately represent the proportions of exposed rock type in modern catchments (Fig. S5; Table S8). These results indicate that, in this setting, detrital samples effectively average the signals of complicating factors such as sediment storage, capture, and variability in bedrock erosion rates. In contrast, we note that although the exposed rock lithologies are generally well-represented by clast compositions, the more durable intrusive rock types are overrepresented in these datasets. We then performed a Kolmogorov-Smirnov statistical analysis of the cumulative detrital zircon U/Pb age distributions to test the extent to which catchments are statistically distinct and to identify plausible source catchment(s) for the Pleistocene deposits (Fig. 3).

We subdivide the detrital zircon geochronology age distributions of modern catchments into two main types (Fig. 3). Catchments that drain both the easternmost San Bernardino Mountains and western Little San Bernardino Mountains (Fig. 3, blue lines) and that yield mostly Proterozoic ages (1400–1800 Ma) are statistically similar and comprise the Type I source. These observations are consistent with more extensive exposures of Precambrian gneiss bedrock lithology within these source areas in the western Little San Bernardino Mountains (Fig. 1d). In contrast, drainages of the Whitewater River and a composite Whitewater River + Mission Creek (in the proposed paleogeographic scenario of ref.^18^) are categorized as Type II sources. These are statistically different than Type I drainages, in that they contain mostly Mesozoic ages with fewer Proterozoic ages (<22%, purple lines in Fig. 3).

### Sedimentology and provenance of the Pleistocene deposits

**Figure 4:**
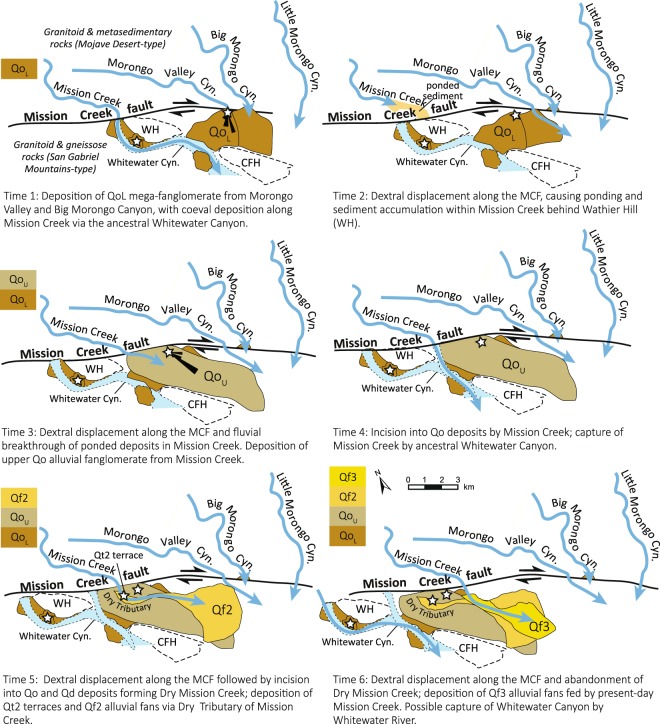
Schematic reconstruction of proposed fault motion and depositional history of the Mid-Pleistocene alluvial fanglomerates along the Mission Creek Fault. White stars show study locations with sedimentary provenance data discussed in the text. Measured paleoflow from imbricated cobbles within the Qo deposits for Times 1 and 3 are shown as black oriented rose diagrams. Paleotopographic highs within the Morongo block outlined as dashed bold lines (CFH = Coachella Fanglomerate Hill; WH = Wathier Hill).

We investigated Pleistocene deposits in the Mission Creek Preserve within the channel walls of the Dry Tributary (Fig. 4), where ~220 m of preserved section exposes north-dipping Early Pleistocene Deformed Gravels of Whitewater (Qd) unconformably overlain by flat-lying Mid-Late Pleistocene Cabezon Formation (Qo) (Fig. 2c). Younger Late Pleistocene deposits^24^ are incised into the Qd and Qo deposits and form Qt2 terraces and Qf alluvial fan deposits (Fig. 4). Field data include measurement of stratigraphic thicknesses and depositional style of conglomerate and interbedded sandstone, paleocurrent measurements, and rock samples for provenance analysis and geochronology (Fig. S2).

The Qd consists of Early Pleistocene moderately stratified conglomerate and sandstone that is interpreted as distal alluvial fan and ephemeral fluvial deposits^37^. Orientations of cobble imbricates within Qd indicate SW and SSE paleoflow directions, confirming an original drainage source to the NE-NNW. Clasts compositions of Qd are mostly monzonite, granodiorite, biotite gneiss, and hornblende diorite with lesser amounts of phyllite and marble.

In contrast, the overlying Qo consists of sub-angular to sub-rounded megabreccia fanglomerate (lower Qo) and upward fining weakly stratified cobble to pebble conglomerate (upper Qo). Orientations of cobble imbricates indicate SW and SSE paleoflow directions in the Early Pleistocene to Late Pleistocene deposits, confirming an original drainage source to the NE-NNW. The upper Qo displays a conspicuous upsection appearance of metasedimentary clasts – such as phyllite, quartzite, and marble – and volcanic clasts (Fig. 2b). Notably, we observe this compositional change in rock type to correspond with an upsection change in lithofacies, from very coarse-grained and poorly sorted megablock debris flow deposits of the lower Qo that are rich in biotite gneiss clasts. Upsection, the deposits grade into weakly stratified debris flow and sheet-flood deposits with a higher proportion of metasedimentary components. These observations suggest a changing source-character of the Qo over time, with increasing sourcing of metasedimentary units and reduced crystalline metamorphic contributions. Modal sandstone compositions are arkosic to lithic-arkosic within Qo, indicating a tectonic provenance of dissected magmatic arc and basement uplift, consistent with derivation from plutons of the Sierra Nevada batholith and metamorphic basement (Fig. S2).

Late Pleistocene terrace fill in Dry Tributary, Qt2, consists of weakly stratified, moderately sorted pebble to cobble conglomerate alluvial deposits (Fig. 4). Modal clast counts from these deposits yield metasedimentary clasts, diorite porphyry, as well as the other igneous rock types (Fig. 2b). Qt2 terraces are formed within the hillslope of Qd and Qo deposits and are interpreted to correlate with Qf2 alluvial fans located at the mouth of the Dry Tributary.

The detrital zircon U/Pb age distributions of the Late Pleistocene Qt2 terrace fill and Cabezon fanglomerate (Qo) are both characterized by mostly Mesoproterozoic ages, nearly equal proportions of Jurassic and Cretaceous ages, and few but noteworthy Paleozoic ages (Fig. 3). In contrast, Qd deposits contain mostly Jurassic and Cretaceous ages with fewer (15–30%) Proterozoic ages. Lastly, we note that the Qd deposits consist of dominantly Mesozoic ages and are not a statistical match to any catchments (Table 1), reflecting a different source outside of our immediate study area, the source is likely located in the eastern Little San Bernardino Mountains where the Mesozoic batholith is extensively and more deeply exposed. Most importantly our K-S statistical analysis of these deposits in comparison to the modern catchments shows that (1) the upper Qo is statistically compatible with a Type I source, with closest affinity to Mission Creek; (2) terrace deposits Qt2 within Dry Tributary are also compatible with Type I source; and (3) neither Whitewater River nor composite Whitewater + Mission Creek are compatible with any of the Pleistocene deposits (Fig. 3).

**Table 1:** Kolmogorov-Smirnoff (K-S) statistics from comparison of detrital zircon U/Pb age distributions between Quaternary deposits and modern source drainages.

Formation	Sample		WWR + MC	WWR	CF	MC	MVC	BMC	LMC
			n = 284	n = 183	n = 100	n = 101	n = 78	n = 96	n = 285
Qt2 terrace fill	MCP-25	n = 278	0.003	0.000	0.000	**0.070**	**0.191**	**0.352**	**0.602**
Cabezon Fanglomerate (Qo)	MCP-6	n = 95	0.000	0.000	0.005	**0.104**	0.005	0.042	0.000
	MCP-7	n = 197	0.000	0.000	0.000	**0.577**	**0.054**	0.042	0.002
Deformed gravels of Whitewater (Qd)	MCP-8	n = 160	0.000	0.000	0.000	0.000	0.000	0.000	0.000
	MCP-22	n = 273	0.000	0.001	0.000	0.000	0.000	0.000	0.000

For samples that yield K-S P-values less than 0.05 (95% confidence), values shown in bold, the two distributions are statistically equivalent. Results show that Qo is statistically compatible with Mission Creek, and to a lesser extent, Morongo Valley Canyon. CF = Catclaw Flat; BMC = Big Morongo Canyon; LMC = Little Morongo Canyon; MC = Mission Creek; MVC = Morongo Valley Canyon; WWR = Whitewater River.

## Discussion and Conclusions

### Reconstructed sediment sources in Mission Creek and Morongo Valley Canyon

Our integrated sedimentary provenance and sedimentology datasets from the Cabezon Fanglomerate (Qo) deposits show that the ancestral Mission Creek and Morongo Valley Canyon are the best potential sources for the Mid-Late Pleistocene fanglomerates. These findings are in contrast to previous work that suggested they were derived from a Whitewater-Mission Creek river system along the fault^18,24^. Based on detrital zircon U/Pb geochronology results, the Qo and Qt2 terrace deposits are most compatible with Type I sources (P-value 0.05–0.58) (Fig. 4). They are statistically different from the Whitewater Canyon detrital signature, which is dominated by Cretaceous age components and a higher plagioclase/K-feldspar ratio. These results are further strengthened by an analysis of the conglomerate clast compositions (Table S4). The Qt2 and Qo deposits (Fig. 2b,c, respectively) are similar in types and abundances of plutonic, metamorphic, and metasedimentary rock types (quartzite, marble, phyllite) to the source areas of Mission Creek and Morongo Valley Canyon (Fig. 2a), whereas in Whitewater River drainages (Fig. 2a,c), these metasedimentary rock types are absent. The Mesozoic zircon ages in these sediments correspond to batholithic intrusive rocks, Neoproterozoic – Paleozoic ages are derived from recycled metasedimentary sources; and Mesoproterozoic ages correspond to high-grade metamorphic units exposed in the San Bernardino and Little San Bernardino Mountains^25,27,38^. We also note that the Jurassic zircon U/Pb age peak covaries with the abundance of hornblende diorite clasts, a rock type that is exposed in the Little San Bernardino Mountains and not present in the San Bernardino Mountains.

These findings show that 1) the basal megabreccia of Qo shares a close compositional and detrital U/Pb geochronological affinity to the underlying Qd (Fig. 3), indicating that early deposition of Qo may have been derived from a similar source to Qd and/or sedimentary reworking of Qd. The very coarse-grained nature also reflects more proximal deposition possible due to tectonic uplift of upland source areas. 2) There is close similarity in clast composition between lower Qo and Big Morongo Valley and Morongo Valley Canyon, with abundant gneiss clasts relative to other drainages. 3) The clast and detrital zircon provenance signature of Qo strata shows evolving sources over time, where lower Qo is best matched with Morongo Valley Canyon, and upper Qo (MCP-7 and MCP-6) is best matched to Mission Creek (Table 1). Both are considered Type II source areas, however, and we suggest that this difference reflects changing source area as the alluvial fan depozone is translated westward as in a conveyer belt model^39^.

Taken together, integrated provenance analysis and comparison between exposed bedrock lithology, modern catchments, and Pleistocene sedimentary deposits supports a Mission Creek source for the Cabezon Fanglomerate and Qt2 deposits and precludes Whitewater River as a viable source. These data require significant post-Mid to Late Pleistocene displacement along the MCF since deposition of the fanglomerates and Qt2 deposits. Presently, kinematic models for the region infer the MCF to be inactive along the restraining bend because the Cabezon Fanglomerate and Qf2 deposits are interpreted to be sourced from Whitewater River. This interpretation would require the ancestral Whitewater River to have tapped the metasedimentary units in the northern San Bernardino Mountains until ~40 ka^24^, followed by southward drainage divide migration that is inconsistent with geomorphic observations of northward headward erosion patterns and low erosion rates as shown by catchment-wide ^10^Be cosmogenic nuclide dating (Fig. 1c). Although we are unable to rule out some changes in drainage patterns since ~500–100 ka, we favor a source interpretation which assumes relative stability of the drainage basins and source areas across the timescale of interest.

We propose an alternative model for alluvial fan sedimentation and strike-slip faulting along the MCF during the last 500 kyr of plate boundary evolution (Fig. 4). At Time 1, the lower Qo (Qo_L_) mega-conglomerate was shed near the mouth of Morongo Valley Canyon and/or Big Morongo Canyon (Fig. 4). The high proportion of biotite gneiss clasts and diorite, with lesser plutonic and metasedimentary components, fit this source area. By Time 2, dextral fault motion along the MCF has displaced the Coachella Valley block, resulting in topographic damming of Mission Creek by the ancestral Wathier Hill and sediment ponding within Mission Creek canyon north of the fault (Fig. 4). During Time 3, continued dextral faulting reopened the southern flow of Mission Creek, causing sediment flux to the basin floor and deposition of the alluvial fan deposits of the upper Qo (Qo_U_) (Fig. 4, Time 2). Subsequent incision of Qo during Time 4 could have resulted in capture of Mission Creek by the ancestral Whitewater Canyon, along a bedrock or structural boundary with the Deformed Gravels of Whitewater paleotopographic high (Fig. 4). By Time 5, continued dextral fault motion resulted in westward displacement of the Qo deposits and incision of Dry Tributary paleodrainage by Mission Creek, forming Qf2 alluvial fans and Qt2 terrace deposits, respectively. Lastly, in Time 6, dextral fault motion resulted in the establishment of Mission Creek in its modern position, resulting in Qf3 and modern alluvial fan deposition.

### Implications for young fault displacement and the San Andreas Fault plate boundary

The alluvial fan reconstructions, based on our integrated provenance data, allow us to provide an alternative interpretation of (1) the PA-NA plate boundary at this latitude and the (2) role of the MCF as a major plate boundary structure (Fig. 4). In contrast to previous studies suggesting this segment of the fault is inactive since the Mid to Late Pleistocene at this latitude^5,18,21,24^, we recognize evidence consistent with continued displacement on this fault of ~2.4–2.5 km since the Late Pleistocene. Figure 4 (Time 5–6) shows the Dry Tributary of Mission Creek right-laterally offset by ~2.4–2.5 km. This measured offset is based on the interpretation that initial incision of the Dry Tributary of Mission Creek must have occurred prior to Mission Creek supplying Late Pleistocene alluvial fan deposits through its channel walls.

This sediment routing reconstruction is broadly consistent with previous studies^18^, except that we interpret Dry Tributary to have been structurally offset since its initial incision and deposition at the mouth of the ancestral Mission Creek based on the following: (1) Our new provenance data show that both Qo and Qt2 alluvial deposits are most likely derived from Mission Creek. (2) Dry Tributary is incised into Qo and Qd deposits. (3) Finally, Late Pleistocene fans, Qf2, are absent near the fault at the mouth of Mission Creek (Fig. 4, Time 6) and are only present – with well-preserved alluvial geomorphology – near the mouth of the Dry Tributary (Fig. 4, Time 5), requiring these deposits to have been transported through Dry Tributary during alluvial fan deposition (Fig. 4. Time 5). Alternatively, any Qf2 sedimentation at the mouth of Mission Creek has since been removed by erosion.

The diffusive alluvial depositional nature of Qo, combined with lack of robust age control on the timing of Pleistocene sedimentation precludes us from calculating a reliable slip rate at this time. However, the incisional geomorphology of Dry Tributary and deposition of Qt2 terrace fill within its canyon walls allows us to estimate a maximum slip rate for the MCF since incision of the Dry Tributary into Qo (Fig. 4, Time 4). Buried sand samples of Qf2 (Fig. 4, Time 5; 4–5 m depth collection depth) yield Infra-Red Stimulated Luminescence (IRSL) dates of 95^+15^/_−15_ ka and 106^+15^/_−15_ ka^18^ that constrain the timing of sedimentary burial within the Dry Tributary^18,24^. Combining the published IRSL dates of Qf2 with the 2.4–2.5 km offset (described above) of the Dry Tributary indicate maximum slip rates along the MCF of up to ~20–30 mm yr^−1^.

These maximum rates for the MCF are comparable to the present-day geodetic slip rate for the southern San Andreas Fault (~23 mm yr^−1^)^22,40–43^ and suggest that the MCF, once the principal structure responsible for ~90 km of total displacement since ca. 27 Ma^6^, remained an active fault into Late Quaternary time. Our findings highlight renewed interest and reevaluation of the young faulting histories along the Mission Creek and Mill Creek strands^44^. Moreover, recently published work on the Banning strand (Fig. 1b), thought to be the dominant active plate boundary structure within the San Gorgonio Pass area, reports low Holocene slip rates of 4–5 mm yr^−1^ and suggests the Banning strand accommodates less Holocene slip than previously thought^23^. More work is needed to better constrain the slip rate for the MCF into Holocene timescales^45^, however, we note that evidence for potential Holocene (?) slip is observed by the presence of three channels that are deflected and offset right-laterally by ~50 m along the MCF at this latitude (Fig. S6). These displaced channels, as of yet undated, are incised into upper Qo deposits and exhibit shutter ridges that topographically block downstream channel flow.

These results and our provenance analysis allow for the possibility that the MCF likely remains the primary plate boundary structure at this latitude. Accordingly, these observations and interpretations may further signify active faulting along the MCF and require revisions to fault kinematic models of strain partitioning and seismic risk assessment along the southern San Andreas Fault^11,46^. We hypothesize that continuous faulting history along the MCF suggests that transform plate boundaries like the San Andreas Fault system may remain stable and long-lived, and deformation may not evolve across different strands as rapidly as suggested by existing evolution models for these fault systems^6,13,47^. Finally, we show that in tectonically active settings with lithologically and geochronologically distinct source areas, detrital provenance analysis deposits can constrain possible fault slip reconstructions, even across Quaternary timescales.

## Methods

Methods, including statements of data availability and references, are available in the online version of this paper. All data generated or analyzed during this study are included in this published article (and its Supplementary Information files).

### Modal analysis of clast compositions

Clast compositional information was collected from twenty sampling stations, including the active drainages and Quaternary deposits (Table S1). To minimize bias toward more durable clast types, we used the area counting technique^48^ for all cobble-sized clasts (between 64 mm and 256 mm in diameter) until 100 counts were reached for each station. We report normalized compositions from twelve diagnostic clast lithologic compositions, Biotite Gneiss, Deformed Granite, Amphibolite and Mafic Schist, Monzonite, Pink K‐feldspar monzonite, Granite and Granodiorite, Diorite, Coarse diorite, Volcanic, Quartzite, Phyllite, and Marble. Recalculated data are shown in Tables S2 and S3 for modern drainages and Quaternary deposits, respectively.

### Detrital zircon U/Pb geochronology

Detrital zircons were extracted from ~5 kg medium-grained sandstone hand-samples or unconsolidated sand using conventional magnetic and density separation techniques. Final zircon concentrates were mounted on tape in epoxy resin, polished to expose the interior of the grain, and imaged with backscattered electron microscopy and cathodoluminescence (CL). Uranium, thorium, and lead isotopes were measured by laser ablation inductively coupled plasma mass spectrometry at the LaserChron Center at the University of Arizona. The analyses involve ablation of zircon with a Photon Machines Analyte G2 excimer laser using a spot diameter of 30 µm. Detrital zircons were randomly analyzed from a linear swath of grains across the sample mount to minimize sampling bias in characterizing all detrital populations. The analytical data are reported in Table S5. Preferred calculated U/Pb ages use the ^204^Pb corrected ^206^Pb/^238^U ratio for <1.0 Ga grains and the ^204^Pb corrected ^206^Pb/^207^Pb ratio for >1 Ga grains. Uncertainties shown in these tables are at the 1σ level, and include only measurement errors. Analyses that are >20% discordant (by comparison of ^206^Pb/^238^U and ^206^Pb/^207^Pb ages) or >5% reverse discordant are were excluded from provenance interpretations. The resulting interpreted ages are shown on Pb*/U concordia diagrams (Fig. S3) and relative age-probability diagrams using the routines in Isoplot^49^ (Fig. S4). The age-probability diagrams show each age and its uncertainty (for measurement error only) as a normal distribution, and sum all ages from a sample into a single curve. Full analytical methods, results, and statistical analysis of all detrital geochronology data are reported in Supplementary Information.

### ^10^Be cosmogenic nuclide data from modern catchments

All ^10^Be isotope samples were processed at the Cosmogenic Radionuclide Target Preparation Facility at Stanford University and analyzed at the Center for Accelerated rated Mass Spectrometry at the Lawrence Livermore National Laboratory. In the field, approximately 2 kilograms of sand were collected from the mouth of modern drainages along the Little San Bernardino Mountains and San Bernardino Mountains (Fig. 1). These sand samples were sieved to a grain size range of 250 to 500 µm, and then leached through a series of 2–3% HF acid to isolate the grains of quartz, the beryllium-bearing mineral^50^. Following quartz separation and purification, ^9^Be was added to the sample as a spike to determine the amount of ^10^Be naturally present in the sample. Beryllium was extracted from the sample using ion chromatography and subsequently converted to beryllium oxide^50,51^, which was then mixed with powdered niobium and targeted for accelerator mass spectrometry. The denudation rates were calculated using the CRONUS Age Calculator V3^52^ available at, http://hess.ess.washington.edu/math/) (Table S8).

## Electronic supplementary material

Supplementary Information

Dataset 1
