# Supplementary material for: Sedimentary signals of recent faulting along an old strand of the San Andreas Fault, USA
Source: Sci Rep. 2018 Aug 14;8:12132. doi: 10.1038/s41598-018-30622-3 (PMC6092442; doi:10.1038/s41598-018-30622-3)
Supplement: Supplementary file 1 — Supplementary Information [file 41598_2018_30622_MOESM1_ESM.pdf]

SUPPLEMENTARY INFORMATION

Sedimentary signals of recent faulting along an old strand of the San Andreas Fault, USA

Julie C. Fosdick[1,2]* and Kimberly Blisniuk[3]

[1]Department of Geography, University of Connecticut, Storrs, CT 06269, USA
[2]Center for Integrative Geosciences, University of Connecticut, Storrs, CT 06269, USA
[3]Department of Geology, San Jose State University, San Jose, CA 95192, USA

*Correspondence to julie.fosdick@uconn.edu

1. Sedimentology and stratigraphy

We conducted field mapping and sedimentary facies analysis of a ~220 m thick composite measured stratigraphic section, here called the Sagebrush Section, through the tilted Deformed Gravel of Whitewater (Qd) and overlying sub-horizontal Cabezon Fanglomerate (Qo), located in the Mission Creek Preserve (**Figure S1**). Lithological descriptions, bed thicknesses, facies, and sedimentary structures were described at decimeter scale (**Figure S1**). Where preserved, paleoflow directions were determined from the orientations of imbricated conglomerate clasts. Sedimentary provenance data collected from this section include clast counts, sandstone petrography, and detrital zircon U/Pb geochronology. Lithofacies shown in **Figure S1** are modified from Miall (1978) and DeCelles et al. (2015).

2. Modal analysis of clast compositions

Clast compositional information was collected from twenty sampling stations, including the active drainages and Quaternary deposits (**Table S1**). To minimize bias toward more durable clast types, we used the area counting technique (e.g., Howard, 1993) for all cobble-sized clasts (between 64 mm and 256 mm in diameter) until 100 counts were reached for each station. We report normalized compositions from twelve diagnostic clast lithologic compositions, Biotite Gneiss, Deformed Granite, Amphibolite and Mafic Schist, Monzonite, Pink K-feldspar monzonite, Granite and Granodiorite, Diorite, Coarse diorite, Volcanic, Quartzite, Phyllite, and Marble. These clast types are modified from Sadler et al. (1993) for comparison with published clast compositional data from modern drainages. Recalculated data are shown in **Table S2** and **Table S3** for modern drainages and Quaternary deposits, respectively.

3. Modal analysis of sandstone petrography

Fifteen samples from medium-grained sandstones were collected from the Deformed gravels of Whitewater (Qd), Cabezon Conglomerate (Qo), Qt3 terrace deposits, and active drainages for sandstone petrographic analysis. Each thin section was prepared by Quality Thin Section, LLC., and stained with Alizarin red S to aid in identification of calcic plagioclase and other Ca-rich phases. Samples were point-counted (for 400 grains) following the Gazzi-Dickinson method[5,6] using a Pelcon automated point counting system and a Leica DMZ2700 petrographic light microscope at the University of Connecticut. Grain parameters identified in these point counts are listed in the data repository, and recalculated data are provided in **Table S4**. All samples are classified as arkosic to lithic-arkosic sandstones (after Folk et al., 1980). **Figure S2** presents ternary diagrams for Monocrystalline Quartz, Feldspar-Total Lithics (Qm-F-Lt) data, showing a dissected and transitional

magmatic arc, and to a lesser extent, basement uplift provenance. Total Quartz-Feldspar-Lithics (Qt-F-L) data show a stronger dissected magmatic arc provenance, likely biased by polycrystalline quartz (derived from basement gneisses) contributing to higher total quartz component. Quartz-Plagioclase-K-feldspar (Q-P-K) data, showing variable Q and K data with relatively uniform P content, except for Whitewater River, which has the highest content.

4. U/Pb geochronologic analyses of detrital zircon (Nu HR ICPMS)

Detrital zircons were extracted from ~5 kg medium-grained sandstone hand-samples or unconsolidated sand using standard mineral separation techniques at the ZirChron, LLC. (Tucson, Arizona), including crushing and grinding, fractionation of magnetic minerals with a Frantz isodynamic magnetic separator, and settling through heavy liquids to exclude phases with densities less than 3.3 g/cm^3. Final zircon separates were mounted in epoxy resin together with fragments of the Sri Lanka standard zircon. The mounts were polished to a depth of ~20 µm, imaged, and cleaned prior to isotopic analysis.

U/Pb geochronology of zircons is conducted by laser ablation multicollector inductively coupled plasma mass spectrometry (LA-MC-ICPMS) at the Arizona LaserChron Center [7,8]. The analyses involve ablation of zircon with a Photon Machines Analyte G2 excimer laser using a spot diameter of 30 µm. The ablated material is carried in helium into the plasma source of a Nu HR ICPMS, which is equipped with a flight tube of sufficient width that U, Th, and Pb isotopes are measured simultaneously. All measurements are made in static mode, using Faraday detectors with 3x10^{11} ohm resistors for ^{238}U, ^{232}Th, ^{208}Pb-^{206}Pb, and discrete dynode ion counters for ^{204}Pb and ^{202}Hg. Ion yields are ~0.8 mv per ppm. Each analysis consists of one 15-second integration on peaks with the laser off (for backgrounds), 15 one-second integrations with the laser firing, and a 30 second delay to purge the previous sample and prepare for the next analysis. The ablation pit is ~15 µm in depth.

For each analysis, the errors in determining ^{206}Pb/^{238}U and ^{206}Pb/^{204}Pb result in a measurement error of ~1-2% (at 2σ level) in the ^{206}Pb/^{238}U age. The errors in measurement of ^{206}Pb/^{207}Pb and ^{206}Pb/^{204}Pb also result in ~1-2% (at 2σ level) uncertainty in age for grains that are >1.0 Ga, but are substantially larger for younger grains due to low intensity of the ^{207}Pb signal. For most analyses, the cross-over in precision of ^{206}Pb/^{238}U and ^{206}Pb/^{207}Pb ages occurs at ~1.0 Ga. ^{204}Hg interference with ^{204}Pb is accounted for measurement of ^{202}Hg during laser ablation and subtraction of ^{204}Hg according to the natural ^{202}Hg/^{204}Hg of 4.35. This Hg is correction is not significant for most analyses because our Hg backgrounds are low (generally ~150 cps at mass 204). Common Pb correction is accomplished by using the Hg-corrected ^{204}Pb and assuming an initial Pb composition[9]. Uncertainties of 1.5 for ^{206}Pb/^{204}Pb and 0.3 for ^{207}Pb/^{204}Pb are applied to these compositional values based on the variation in Pb isotopic composition in modern crystalline rocks. Inter-element fractionation of Pb/U is generally ~5%, whereas apparent fractionation of Pb isotopes is generally <0.2%. In-run analysis of fragments of a large zircon crystal (generally every fifth measurement) with known age of 563.5 ± 3.2 Ma (2σ error) is used to correct for this fractionation. The uncertainty resulting from the calibration correction is generally 1-2% (2σ) for both ^{206}Pb/^{207}Pb and ^{206}Pb/^{238}U ages. Concentrations of U and Th are calibrated relative to our Sri Lanka zircon, which contains ~518 ppm of U and 68 ppm Th.

The analytical data are reported in **Table S5**. Preferred calculated U-Pb ages use the ^{204}Pb corrected ^{206}Pb/^{238}U ratio for <1.0 Ga grains and the ^{204}Pb corrected ^{206}Pb/^{207}Pb ratio for >1 Ga grains. Uncertainties shown in these tables are at the 1σ level, and include only measurement errors. Analyses that are >20% discordant (by comparison of ^{206}Pb/^{238}U and ^{206}Pb/^{207}Pb ages) or >5%

reverse discordant are were excluded from provenance interpretations. The resulting interpreted ages are shown on Pb*/U concordia diagrams (**Figure S3**) and relative age-probability diagrams using the routines in Isoplot[10] (**Figure S4**). The age-probability diagrams show each age and its uncertainty (for measurement error only) as a normal distribution, and sum all ages from a sample into a single curve.

5. Pearson Chi-squared Statistical Analysis of Modern Catchments

We evaluated the probability of the detrital clast types and detrital zircon U/Pb ages sourced from each catchment by performing a nonparametric statistical analysis on the clast populations from Quaternary deposits and modern catchments.
First, we grouped the twelve observed clast types into three genetically related categories: (1) crystalline metamorphic, (2) intrusive, and (3) metasedimentary, sedimentary, and volcanic. We applied the Pearson chi-square (χ^2) statistic[11] as a measure of the goodness of fit between the observed clast lithology categorical data, O_i, (i.e., the Qo sample) and the expected clast lithology distribution based on exposed bedrock, E_i, (i.e. the catchment source) for n = 100 total counts.

$$\chi^2_{c-1} = \Sigma\, (O_i - E_i)^2/E_i$$

Calculated χ^2 statistics were compared to a P-value = 9.488 for 0.05 level of significance and two degrees of freedom (**Table S6**). For samples that yield χ^2 values less than the P-value, the null hypothesis is satisfied and we interpret the observed clast or zircon U/Pb age distribution as equivalent to the predicted rock types exposed bedrock lithology.

The χ^2 results show that observed detrital datasets satisfy the Chi-squared goodness of fit test and match the expected distributions, with χ^2 values between 0.011-13.530. Only the observed clast distribution of Little Morongo Canyon does not match the predicted distribution, possibly due to higher metasedimentary clasts derived from Morongo Valley. We note that clast type categories that contribute to largest Pearson residuals are the metamorphic lithology (biotite gneiss, amphibolite), and the metasedimentary lithologies, as observed qualitative differences. **Figure S5** compares the proportions of rock types based on exposed bedrock lithology, observed detrital zircon U/Pb ages, and clast types. See manuscript text for discussion.

We also performed the χ^2 statistical goodness-of-fit test between the modern catchments and the Quaternary deposits. **Table S7** reports the χ^2 results and shows intraformational variability, as expected given the variability in clast type durability and preservation during transport. The Qt2 sample distribution is only compatible with Mission Creek. The Cabezon Fanglomerate yields only one sample with similarity with Mission Creek. The Wathier Hill Qo sample distribution matches Whitewater, Catclaw Flat, Morongo Valley Canyon, Big Morongo Canyon, and little Morongo Canyon.

6. Kolmogorov-Smirnoff Statistical Analysis

We compare the detrital zircon U/Pb age distributions from the Quaternary deposits with potential source areas using the Kolmogorov-Smirnoff (K-S) statistic on the calculated cumulative zircon He date distributions as a goodness-of-fit criterion[12,13]. **Table 1** shows the results of our analysis for comparing Quaternary deposits with modern drainages. Highlighted results indicate two samples are statistically drawn from the same population, here used as a measure of compatibility between source area and Quaternary deposits. The K-S test is sensitive to overall differences in both age and

magnitude of sample cumulative distributions, does not assume normal distributions, and is commonly used for comparing age distributions in detrital studies[14,15]. Here we use it to evaluate similarity between age distributions in catchments and offset alluvial fan deposits. As constructed, the K-S test is only capable of demonstrating that two distributions are dissimilar if the K-S statistic falls below a specified confidence level. For example, 95% confidence in the K-S test corresponds to a value of the K-S statistic of 0.05. Values of the statistic higher than 0.05 are indistinguishable at the 95% confidence level.

7. ^{10}Be cosmogenic nuclide data from modern catchments

All ^{10}Be isotope samples were processed at the Cosmogenic Radionuclide Target Preparation Facility at Stanford University and analyzed at the Center for Accelerated rated Mass Spectrometry at the Lawrence Livermore National Laboratory. In the field, approximately 2 kilograms of sand were collected from the mouth of modern drainages along the Little San Bernardino Mountains and San Bernardino Mountains (**Fig. 1**). These sand samples were sieved to a grain size range of 250 to 500 µm, and then leached through a series of 2-3% HF acid to isolate the grains of quartz, the beryllium-bearing mineral[16]. Following quartz separation and purification, ^{9}Be was added to the sample as a spike to determine the amount of ^{10}Be naturally present in the sample. Beryllium was extracted from the sample using ion chromatography and subsequently converted to beryllium oxide[16,17], which was then mixed with powdered niobium and targeted for accelerator mass spectrometry. The denudation rates were calculated using the CRONUS Age Calculator V3[18] available at http://hess.ess.washington.edu/math/) (**Table S8**).

8. References

1. Miall, A. D. Lithofacies types and vertical profile models in braided river deposits: a summary. *Mem. Can. Soc. Pet. Geol.* **5,** 597–600 (1978).

2. DeCelles, P. G. *et al.* The Miocene Arizaro Basin, central Andean hinterland: Response to partial lithosphere removal? *Geol. Soc. Am. Mem.* **212,** 359–386 (2015).

3. Howard, J. L. The statistics of counting clasts in rudites: a review, with examples from the upper Palaeogene of southern California, USA. *Sedimentology* **40,** 157–174 (1993).

4. Sadler, P. M., Demirer, A., West, D. & Hillenbrand, J. M. The Mill Creek Basin, the Potato Sandstone, and fault strands in the San Andreas fault zone south of the San Bernardino Mountains. *Geol. Soc. Am. Mem.* **178,** 289–306 (1993).

5. Ingersoll, R. V. *et al.* The Effect of Grain Size on Detrital Modes: A Test of the Gazzi-Dickinson Point-Counting Method. *SEPM Journal of Sedimentary Research* **Vol. 54,** 103–116 (1984).

6. Dickinson, W. R. in *Provenance of Arenites* (ed. Zuffa, G. G.) 333–361 (NATO Advanced Studies Institute, 1985). doi:10.1007/978-94-017-2809-6_15

7. Gehrels, G. E., Valencia, V. & Pullen, A. in *From Geochronology: Emerging Opportunities, Paleontological Society Short Course* (ed. Olszewski, T.) **12,** 67–76 (The Paleontological Society, 2006).

8. Gehrels, G. E., Valencia, V. A. & Ruiz, J. Enhanced precision, accuracy, efficiency, and spatial resolution of U-Pb ages by laser ablation-multicollector-inductively coupled plasma-mass spectrometry. *Geochemistry, Geophys. Geosystems* **9,** 1–13 (2008).

9. Stacey, J. S. & Kramers, J. D. Approximation of terrestrial lead on a subset of magmatic rocks and isotope evolution by a two-stage model. *Earth Planet. Sci. Lett.* **26,** 207–221 (1975).

10. Ludwig, K. R. User's Manual for Isoplot 3.60 - A Geochronological Toolkit for Microsoft Excel. *Berkeley Geochronol. Cent. Spec. Publ.* **4,** 77 (2008).

11. Dixon, W. J. & Massey, F. J. *Introduction to Statistical Analysis*. (McGraw-Hill, 1957).

12. Lovera, O. M., Grove, M., Kimbrough, D. L. & Abbott, P. L. A method for evaluating basement exhumation histories from closure age distributions of detrital minerals. *J. Geophys. Res.* **104,** 29421–29438 (1999).

13. Cina, S. E. *et al.* Gangdese arc detritus within the eastern Himalayan Neogene foreland basin: Implications for the Neogene evolution of the Yalu-Brahmaputra River system. *Earth Planet. Sci. Lett.* **285,** 150–162 (2009).

14. DeCelles, P. G., Carrapa, B. & Gehrels, G. E. Detrital zircon U-Pb ages provide provenance and chronostratigraphic information from Eocene synorogenic deposits in northwestern Argentina. *Geology* **35,** 323 (2007).

15. Gehrels, G. Detrital Zircon U-Pb Geochronology: Current Methods and New Opportunities. *Tectonics Sediment. Basins* 45–62 (2011). doi:10.1002/9781444347166.ch2

16. Gosse, J. C. & Phillips, F. M. Terrestrial in situ cosmogenic nuclides:theory and application. *Quat. Sci. Rev.* **20,** 1475–1560 (2001).

17. Kohl, C. P. & Nishiizumi, K. Chemical isolation of quartz for measurement of in situ-produced cosmogenic nuclides. *Geochemica Cosmochem. Acta* **56,** 3583–3587 (1992).

18. Balco, G., Stone, J. O., Lifton, N. A. & Dunai, T. J. A complete and easily accessible means of calculating surface exposure ages or erosion rates from 10Be and 26Al measurements. *Quat. Geochronol.* **3,** 174–195 (2008).

19. Folk, R. L. Petrology of the sedimentary rocks. *Geomorphology* 190 (1968). doi:10.1016/0169-555X(91)90027-8

20. Bevis, M. & Hudnut, K. B4 Lidar Project: Airborne Laser Swath Mapping (ALSM) survey of the San Andreas Fault (SAF) system of central and southern California, including the Banning segment of the SAF and the San Jacinto fault system. in *National Center for Airborne Laser Mapping (NCALM), U.S. Geological Survey, the Ohio State University, and the Southern California Integrated GPS Project, distributed by OpenTopography.* (2005). doi:10.5066/F7TQ5ZQ6.

Supplementary Tables

Table S1. Sample locations for detrital zircon U/Pb LA-ICPMS geochronology and sandstone petrography.

Table S2. Modal clast compositional data from active drainages.

Table S3. Modal clast compositional data from Quaternary deposits.

Table S4. Recalculated modal sandstone petrographic point-count data.

Table S5. Detrital zircon U/Pb ICP-MS geochronological data. Detrital zircon U-Pb geochronologic analyses by LA-ICP-MS analysis. The * indicates radiogenic Pb (corrected for common Pb). All errors are reported at the 1σ level.

Table S6. Chi-squared statistics of observed proportion of rock types and associated detrital zircon age categories (crystalline metamorphic, intrusives, metasedimentary/sedimentary) with expected proportions from exposed bedrock lithology. For samples that yield $\chi 2$ values less than the P-value (gray highlight), the null hypothesis is satisfied and the observed and expected distributions are statistically equivalent.

Table S7. Chi-squared statistics of observed proportions of clast lithology from the Quaternary deposits with expected proportions from potential source areas. For samples that yield $\chi 2$ values less than the P-value (gray highlight), the null hypothesis is satisfied and the observed and expected distributions are statistically equivalent.

Table S8. ^{10}Be data from active drainages.

Supplementary Figures

Figure S1. Stratigraphy and sedimentology of the Mission Creek fanglomerates exposed in the Sagebrush section, showing lithofacies, paleoflow measurements, and locations of clast counts and sample for detrital geochronology and sandstone petrography. The tilted Deformed Gravels of Whitewater (Qd) are unconformably overlain by sub-horizontal deposits of the Cabazon Fanglomerate (Qo). **Figure S2.** Sandstone petrographic data from Quaternary deposits and modern rivers showing relative proportions of framework minerals, tectonic provenance fields, and sandstone composition. Refer to **Table S4** for point-counting data. a) Monocrystalline Quartz, Feldspar-Total Lithics (Qm-F-Lt) data show a dissected and transitional magmatic arc, and to a lesser extent, basement uplift provenance. b) Total Quartz-Feldspar-Lithics (Qt-F-L) data show a stronger dissected magmatic arc provenance, likely biased by polycrystalline quarts (derived from basement gneisses) contributing to higher total quartz component. c) Quartz-Feldspar-Rock Fragments (Q-F-R), showing predominantly arkosic-to-lithic-arkosic sandstone compositions (after Folk, 1968). d) Quartz-Plagioclase-K-feldspar (Q-P-K) data, showing variable quartz and K-feldspar and uniformly low plagioclase content, except for Whitewater River, which has the highest relative proportion of plagioclase. Modal sandstone compositions from Qd are characterized by an upsection transition from basement uplift to dissected magmatic arc provenance fields and increase in plutonic character (Fig. S2), consistent with unroofing of the Mesozoic Sierra Nevada batholith and Proterozoic metamorphic basement of the Mojave Province.

Figure S3. Tera-Wasserburg Concordia diagrams for detrital zircon U/Pb data. All plots were made with Isoplot[10]. Ellipses show 2σ uncertainty in ages.

Figure S4. Relative probability distributions of zircon U/Pb ages from the a) modern catchments draining the San Bernardino and Little San Bernardino Mountains, b) Late Pleistocene terrace fill, and c) Mid-Pleistocene deposits measured in the Sagebrush Section. Note break in scale between 400 and 1300 Ma (no zircons of this age range).

Figure S5. Predicted proportions of exposed bedrock lithology, based on contributing areas of drainage basins, and the observed detrital zircon U/Pb age categories and clast types. Observed zircon U/Pb results yield acceptable representations of bedrock lithology, whereas clast types generally over-represent more durable rock types (i.e., Mesozoic intrusives).

Figure S6. High-resolution digital topography resolved from Light Detection and Ranging (LiDAR) data[20] from the Mission Creek Fault near the mouth of Mission Creek (see Fig. 1 for location). Red lines show the location of the north and south splay of the Mission Creek Fault. Note the uplifted planar surface between the two splays. We map and interpret the uplifted planar surface is the result of a left step along the Mission Creek Fault from the north splay to the right splay. Base hillshade was generated with ESRI ArcMap v.10.4.1 software (under fair terms of use https://www.esri.com/en-us/legal/copyright-trademarks).

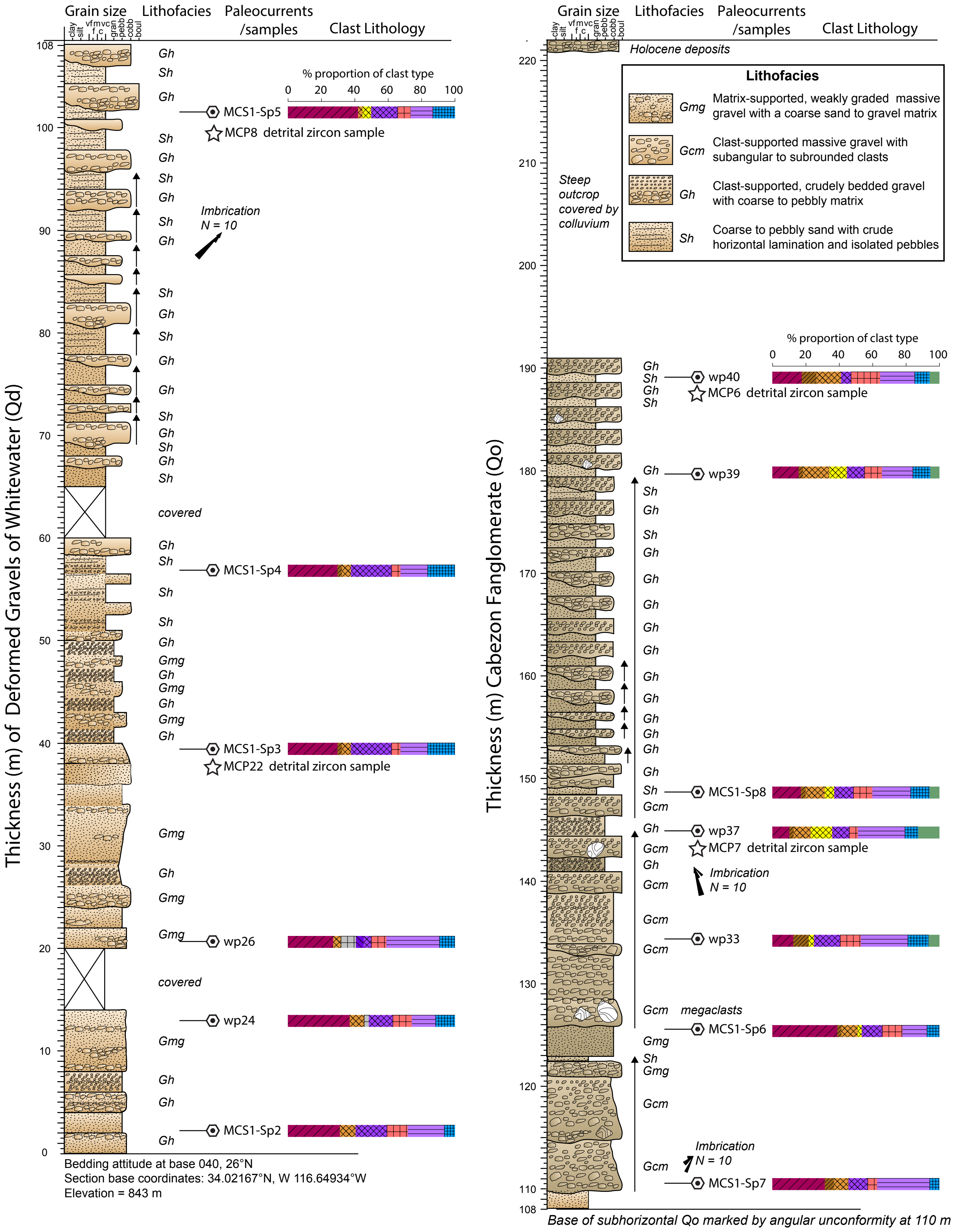

Figure S1. Stratigraphy and sedimentology of the Mission Creek fanglomerates exposed in the Sagebrush section, showing lithofacies, paleoflow measurements, and locations of clast counts and sample for detrital geochronology and sandstone petrography. The tilted Deformed Gravels of Whitewater (Qd) are unconformably overlain by sub-horizontal deposits of the Cabazon Fanglomerate (Qo).

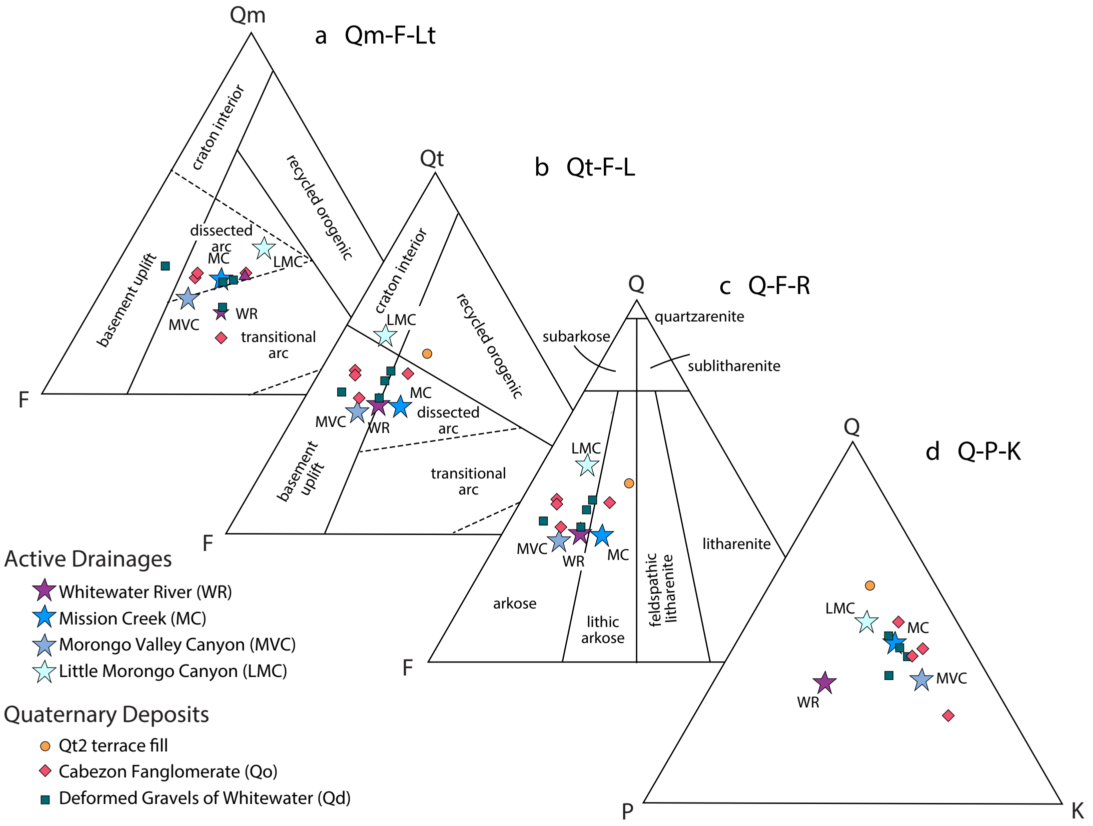

Figure S2. Sandstone petrographic data from Quaternary deposits and modern rivers showing relative proportions of framework minerals, tectonic provenance fields, and sandstone composition. Refer to Table S4 for point-counting data. a) Monocrystalline Quartz, Feldspar-Total Lithics (Qm-F-Lt) data show a dissected and transitional magmatic arc, and to a lesser extent, basement uplift provenance. b) Total Quartz-Feldspar-Lithics (Qt-F-L) data show a stronger dissected magmatic arc provenance, likely biased by polycrystalline quarts (derived from basement gneisses) contributing to higher total quartz component. c) Quartz-Feldspar-Rock Fragments (Q-F-R), showing predominantly arkosic-to-lithic-arkosic sandstone compositions (after Folk, 1968). d) Quartz-Plagioclase-K-feldspar (Q-P-K) data, showing variable quartz and K-feldspar and uniformly low plagioclase content, except for Whitewater River, which has the highest relative proportion of plagioclase. Modal sandstone compositions from Qd are characterized by an upsection transition from basement uplift to dissected magmatic arc provenance fields and increase in plutonic character (Fig. S2), consistent with unroofing of the Mesozoic Sierra Nevada batholith and Proterozoic metamorphic basement of the Mojave Province.

WWR-1 (Whitewater River)

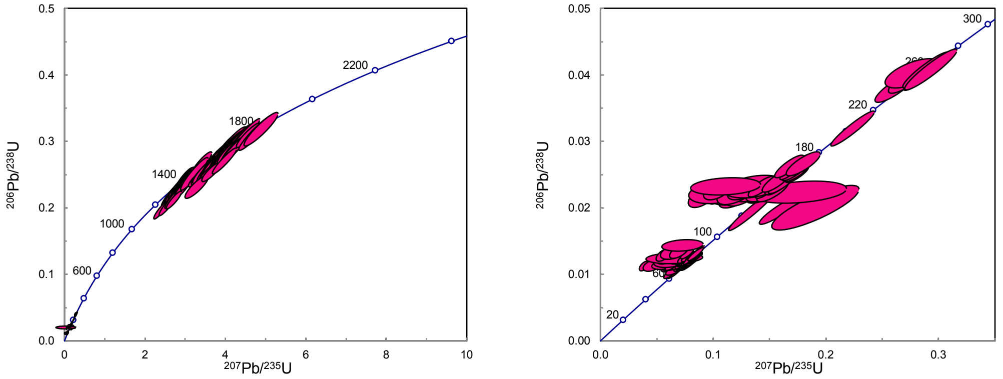

MCP-13 (Catclaw Flat)

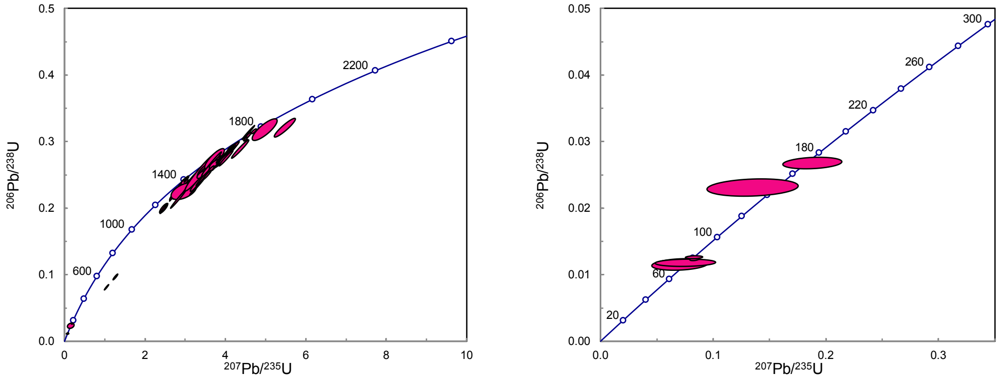

MCP-11 (Mission Canyon Preserve)

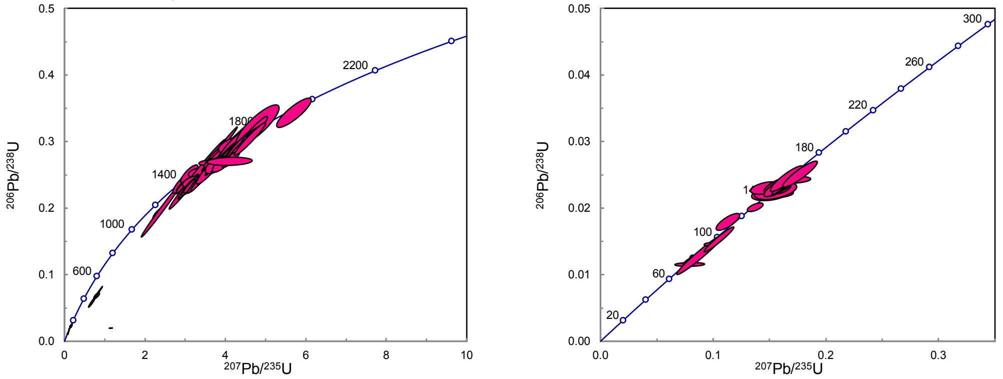

MVC-1 (Morongo Valley Canyon)

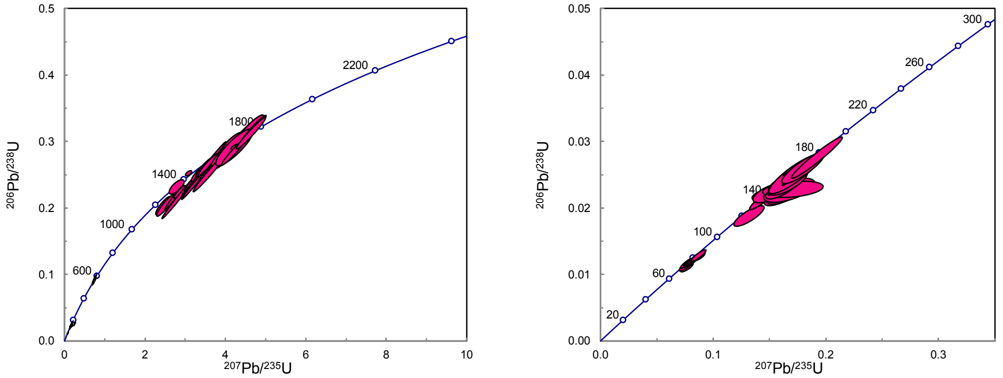

Figure S3. Tera-Wasserburg Concordia diagrams for detrital zircon U/Pb data. All plots were made with Isoplot (Ludwig, 2008). Ellipses show 2σ uncertainty in ages.

BMC (Big Morongo Canyon)

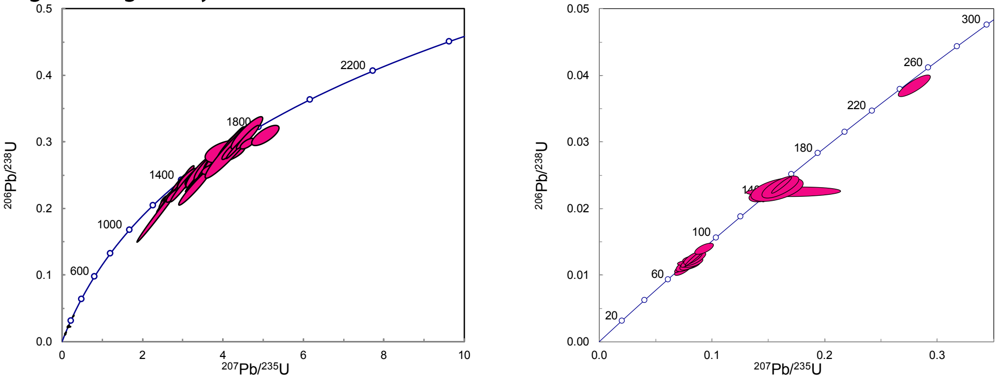

LMC-01 (Little Morongo Canyon)

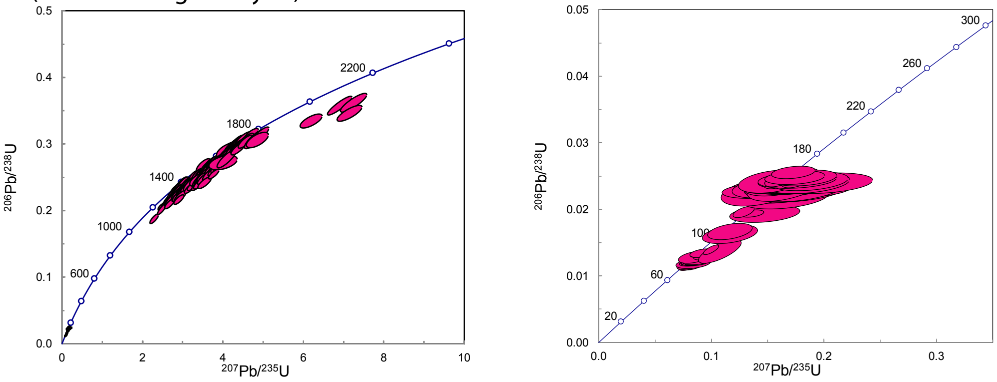

MCP-25 (Qt2 terrace fill; Dry Tributary)

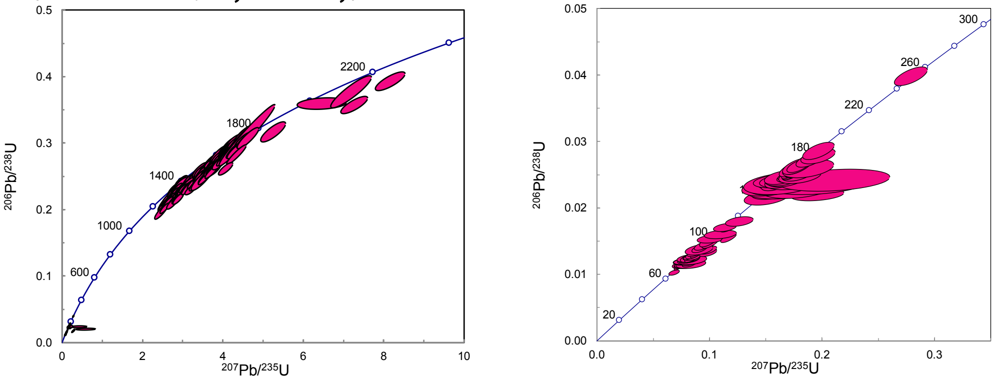

MCP6 (Cabezon Fanglomerate-Qo)

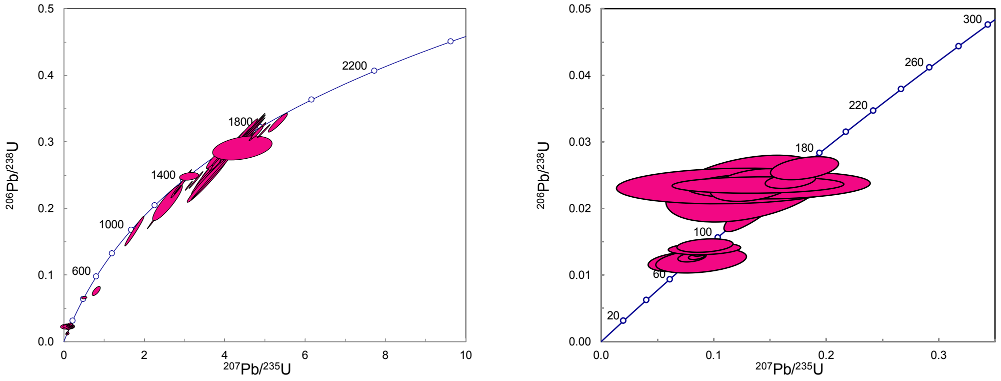

Figure S3 Continued. Detrital zircon U/Pb concordia diagrams.

MCP7 (Cabezon Fanglomerate-Qo)

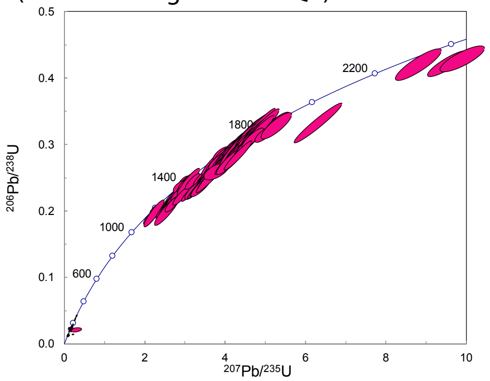

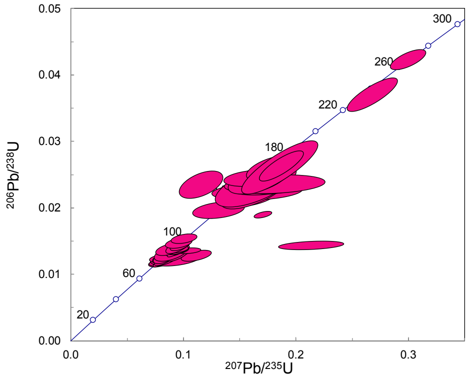

MCP8 (Deformed Gravels of Whitewater-Qd)

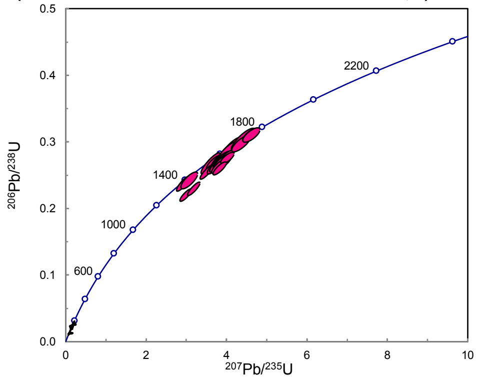

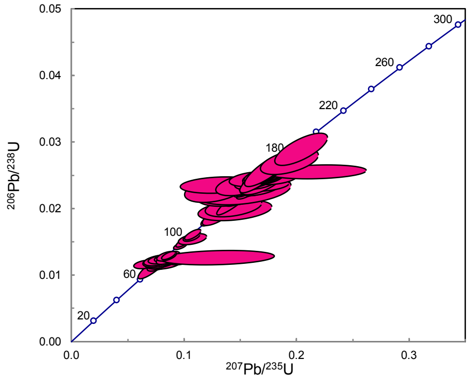

MCP22 (Deformed Gravels of Whitewater-Qd)

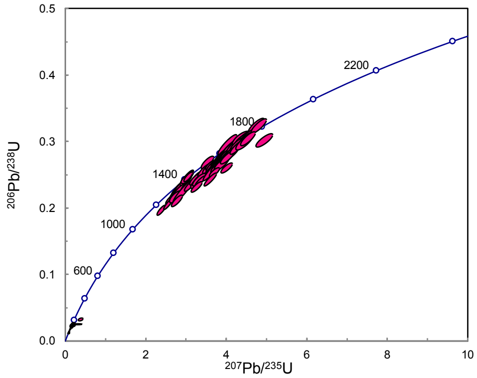

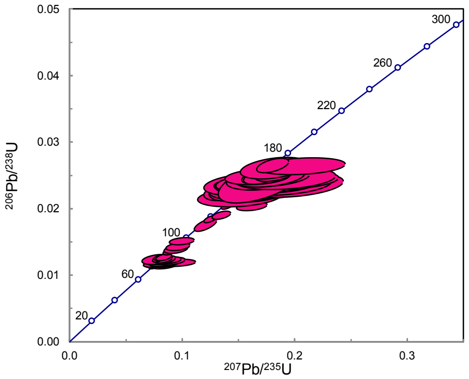

Figure S3 Continued. Detrital zircon U/Pb concordia diagrams.

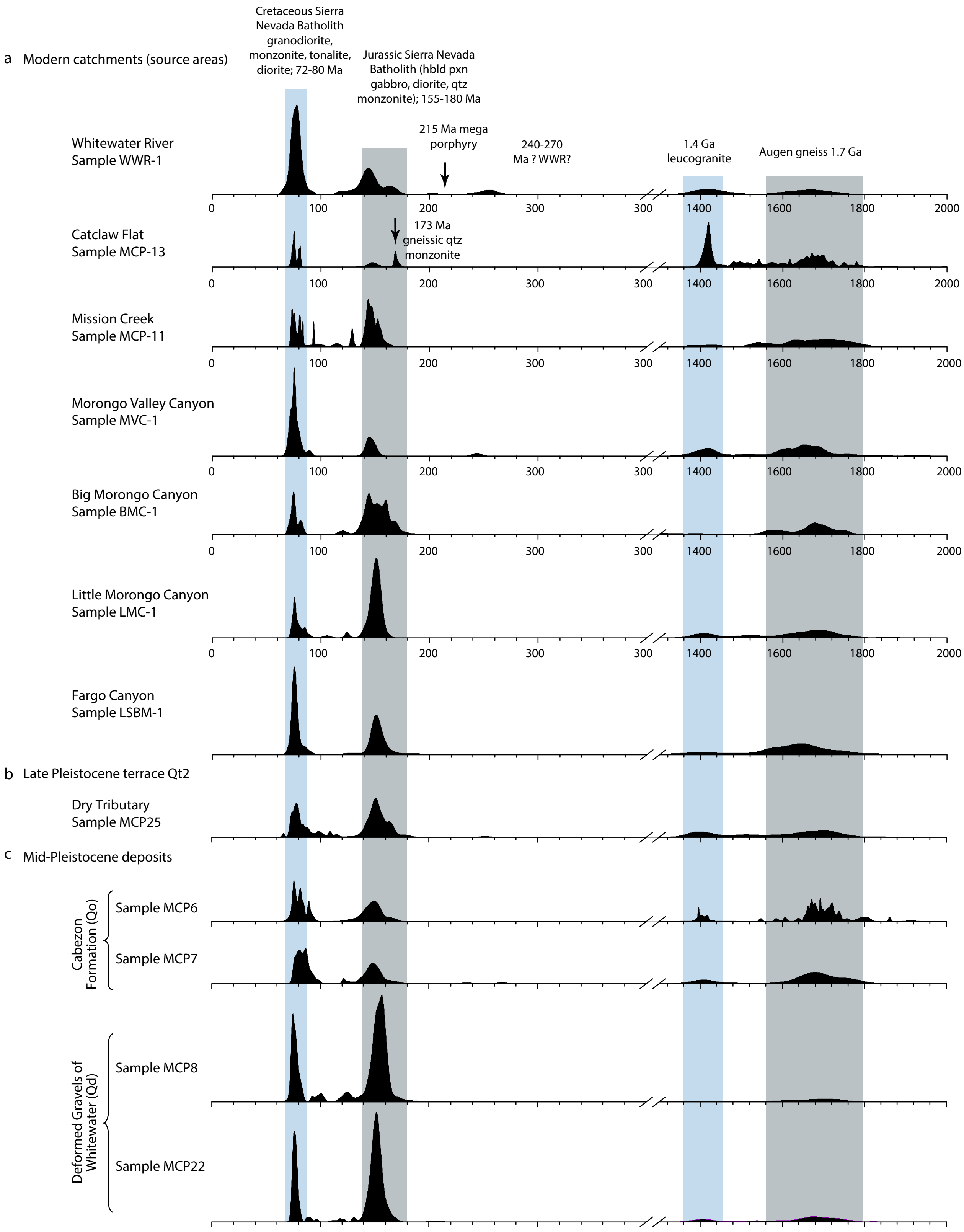

Figure S4. Relative probability distributions of zircon U/Pb ages from the a) modern catchments draining the San Bernardino and Little San Bernardino Mountains, b) Late Pleistocene terrace fill, and c) Mid-Pleistocene deposits measured in the Sagebrush Section. Note break in scale between 400 and 1300 Ma (no zircons of this age range).

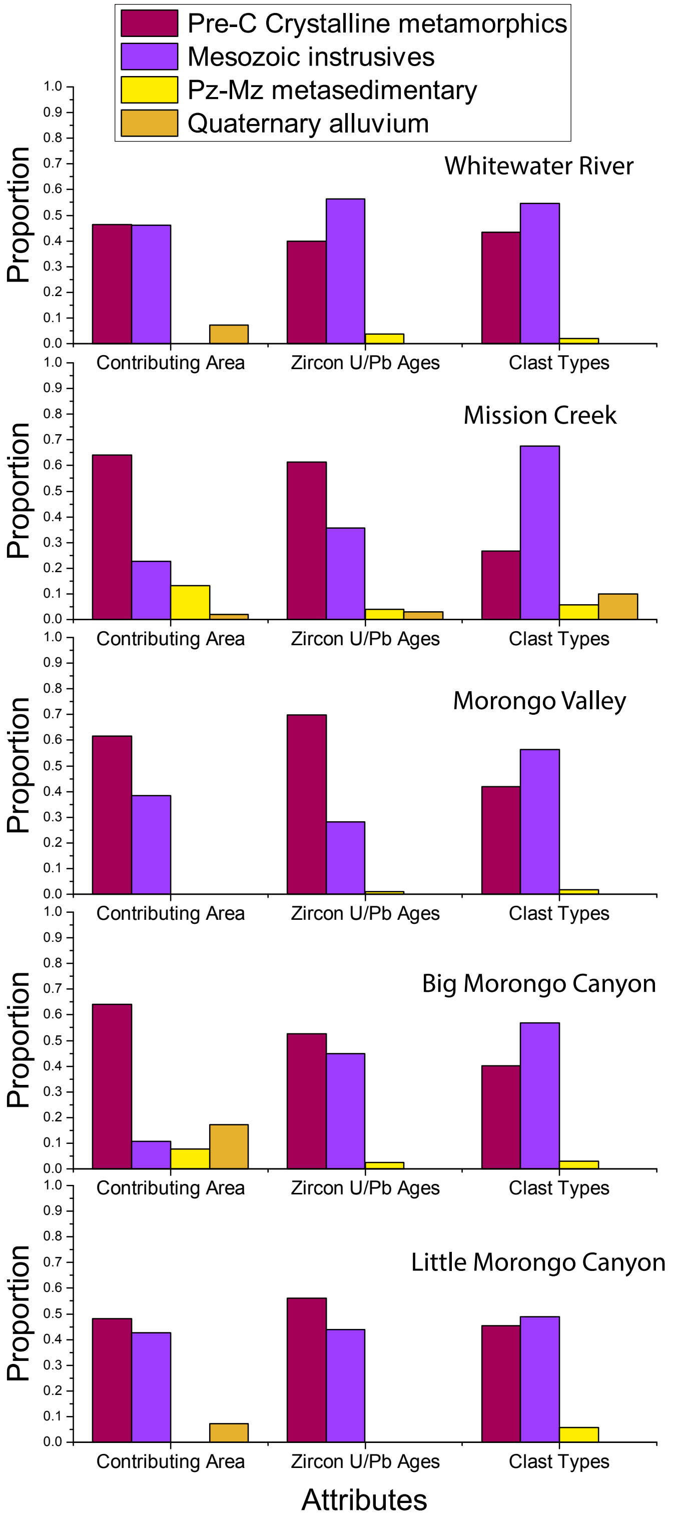

Figure S5. Predicted proportions of exposed bedrock lithology, based on contributing areas of drainage basins, and the observed detrital zircon U/Pb age categories and clast types. Observed zircon U/Pb results yield acceptable representations of bedrock lithology, whereas clast types generally over-represent more durable rock types (i.e., Mesozoic intrusives).

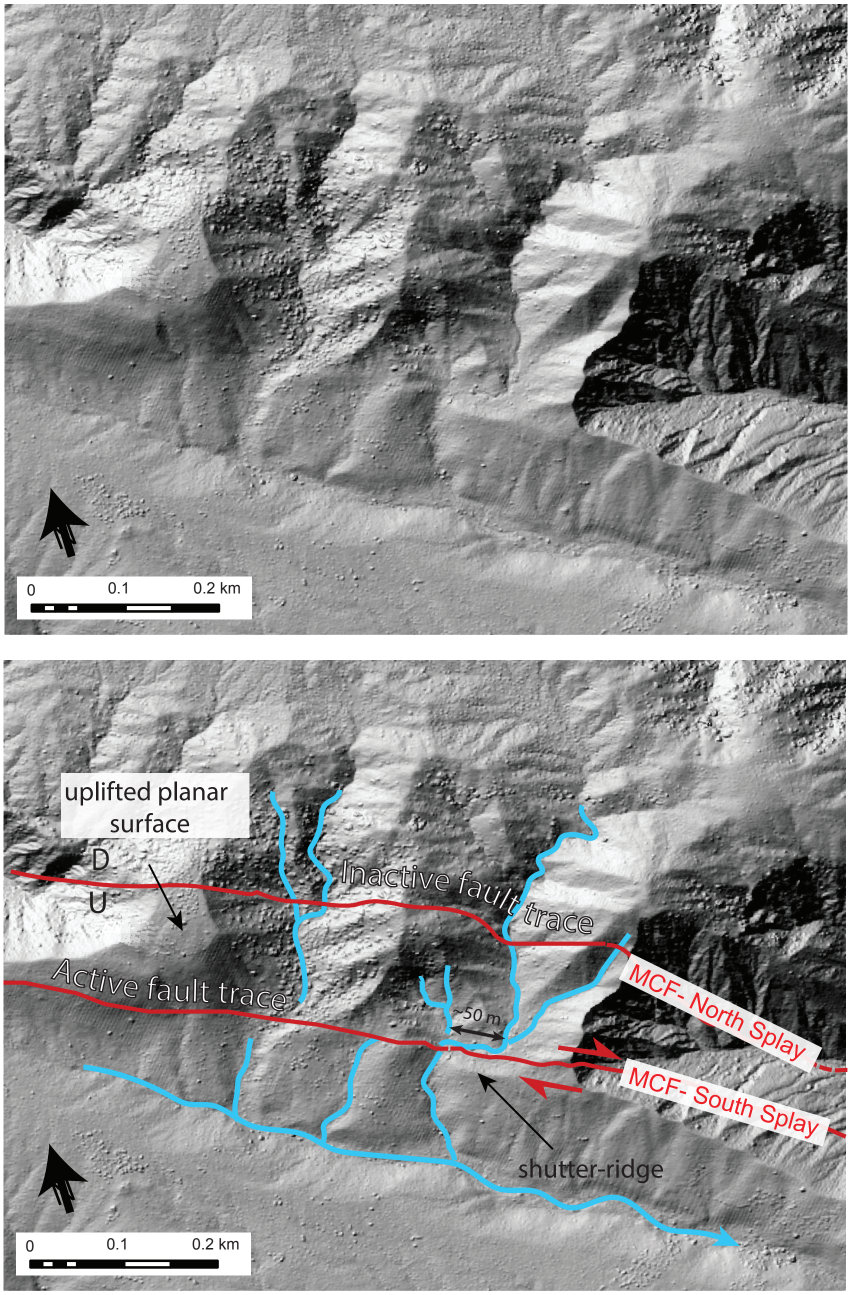

Figure S6. High-resolution digital topography resolved from Light Detection and Ranging (LiDAR) data[20] from the Mission Creek Fault near the mouth of Mission Creek (see Fig. 1 for location). Red lines show the location of the north and south splay of the Mission Creek Fault. Note the uplifted planar surface between the two splays. We map and interpret the uplifted planar surface is the result of a left step along the Mission Creek Fault from the north splay to the right splay. Base hillshade was generated with ESRI ArcMap v.10.4.1 software (under fair terms of use https://www.esri.com/en-us/legal/copyright-trademarks).
